# Repurposing Disulfiram for Cancer Therapy: Mechanistic Insights and Translational Challenges

**DOI:** 10.3390/ijms27177667

**Published:** 2026-08-27

**Authors:** Anna Bilska-Wilkosz, Magdalena Górny, Małgorzata Iciek

**Affiliations:** Chair of Medical Biochemistry, Faculty of Medicine, Jagiellonian University Medical College, Kopernika 7, 31-034 Kraków, Poland; magdalena.gorny@uj.edu.pl (M.G.); malgorzata.iciek@uj.edu.pl (M.I.)

**Keywords:** disulfiram, drug repurposing, diethyldithiocarbamate (DDC), bis(diethyldithiocarbamate)copper(II): Cu(DDC)_2_, DDC/Cu, anticancer therapy, copper ions (Cu^2+^)

## Abstract

Disulfiram (DSF), long used as an aversive agent in alcohol dependence therapy, has recently regained attention as a promising candidate for oncological drug repurposing. After administration, DSF is rapidly reduced to diethyldithiocarbamate (DDC), which, in the presence of Cu^2+^, forms the complex Cu(DDC)_2_. This compound acts as a strong inducer of oxidative stress, an inhibitor of the ubiquitin–proteasome system, and a suppressor of endogenous hydrogen sulfide (H_2_S) synthesis. DSF also modifies protein and non-protein thiol groups, disrupting cancer cell metabolism and promoting apoptosis. Despite robust preclinical evidence, clinical translation remains limited. Key obstacles include DSF’s rapid metabolism, insufficient availability of free copper ions in humans, and the lack of predictive biomarkers capable of identifying responsive patients. Another challenge is DSF’s low oral bioavailability, which prevents the drug from reaching tumor tissue at therapeutically effective concentrations. Consequently, current research focuses on advanced nanocarrier systems designed to protect DSF from premature degradation and ensure its controlled release within the tumor microenvironment. This review summarizes the multifaceted anticancer mechanisms of DSF and discusses biological and pharmacological factors underlying the discrepancies between experimental findings and clinical outcomes.

## 1. Introduction

**Figure 1:**
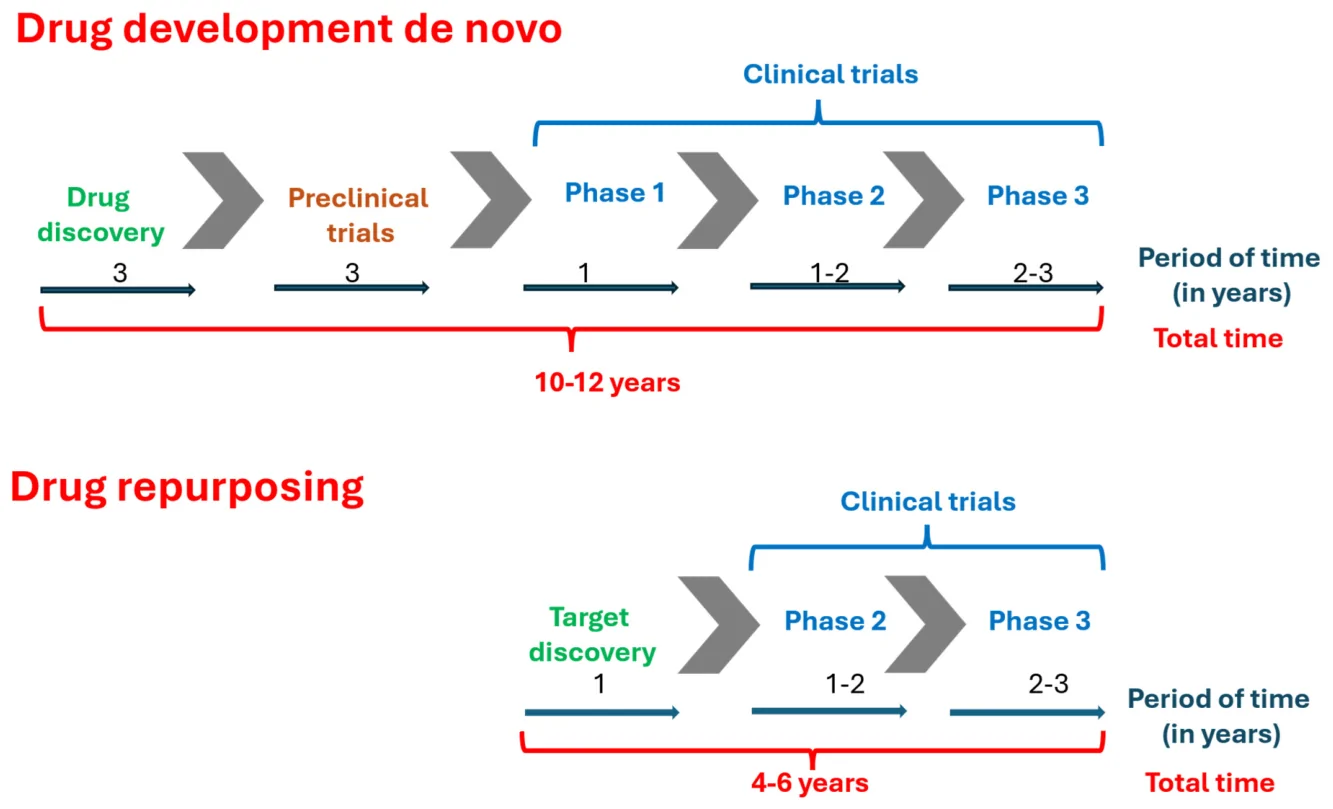
A diagram comparing the number of stages and duration of de novo drug development and drug repurposing. In the case of drug repurposing, preclinical trials (animal studies) and phase 1 of clinical studies are available for existing drugs, and they do not have to be conducted. Drug repurposing begins with new target discovery for an existing drug, followed by phases 2 and 3 of clinical trials. Modified from Park [3].

It has been calculated that to introduce one drug into the market, pharmaceutical companies spend approximately USD 3 billion, and the process itself may take up to 20 years [1]. An attractive alternative to de novo drug design is a strategy called drug repurposing and/or repositioning, defined as the reuse of an active pharmaceutical ingredient currently on the market for a new indication. The history of medicine has many examples of drug repurposing, but most of them occurred solely by chance. Nowadays, however, accidental discoveries have been replaced by a rational, knowledge-based system for searching for new therapeutic applications for known drugs. In addition to experimental methods, computer methods for analyzing biomedical data are used, using modern bio- and chemoinformatic tools. Experimental approaches include targeted screening, cell analysis, animal models, and clinical trials [2]. This strategy is attracting significant interest from pharmaceutical companies because it shortens the time to market and is extremely cost-effective (Figure 1).

The drug repositioning strategy is proving effective, since the list of such repositioned drugs is currently very long and some of the drugs on the list have already obtained marketing authorization for a new indication. In this review, we highlight the potential for repurposing disulfiram (DSF), which has been shown to be effective against diverse cancer types in many animal and in vitro studies, as well as in clinical trials. Recent analyses have further emphasized the importance of rational drug repurposing strategies, including DSF, in modern oncology [4,5].

We note that previous reviews on DSF have typically focused either on its biochemical mechanisms or on early clinical observations. However, none have integrated mechanistic insights with the translational barriers that explain the discrepancy between strong preclinical efficacy and weak clinical outcomes. The present review fills this gap by combining copper-dependent pharmacology, proteasome inhibition, H_2_S signaling, cancer stem cell biology, and the emerging role of nanoformulations with a critical analysis of pharmacokinetic limitations and biomarker deficiencies. This integrated perspective provides an updated and comprehensive explanation of why DSF has not yet succeeded clinically despite its robust mechanistic potential.

As a side note, it is worth mentioning that beyond oncology, DSF also exhibits antibacterial and antiviral activities, including anti-HIV effects [6,7]. This was also confirmed by our research, in which we showed for the first time that diethyldithiocarbamate (DDC), which is the main metabolite of DSF, possesses antibacterial activity against *Ureaplasma* spp., a genus of cell wall-free bacteria [8]. However, these antimicrobial activities of DSF are mechanistically distinct and are not the focus of the present article. We mention them only because they illustrate the pleiotropic nature of the compound.

## 2. Short Story of Disulfiram

**Figure 2:**
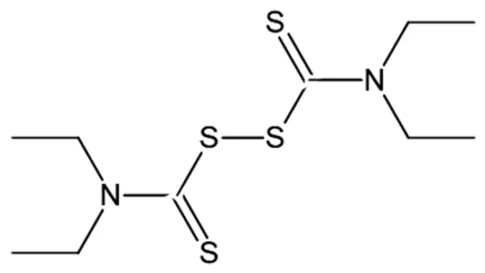
The chemical structure of disulfiram shows that it is a symmetrical disulfide.

Disulfiram (DSF; IUPAC: *N*,*N*-diethyl[(diethylcarbamothioyl)disulfanyl]carbothioamide]; trade name Antabuse) is tetraethylthiuram disulfide. It is a dimeric molecule in which two diethyldithiocarbamate (DDC) moieties are linked through a sulfur atom, forming a disulfide bond. Its structure is shown in Figure 2.

Its history began over 140 years ago. It was then a peak period in the history of organic synthesis, when chemists produced new compounds almost en masse. DSF, a new compound synthesized from thiocarbamide in 1881, seemed to be just another such compound and did not attract much attention [9]. It is an odorless, practically tasteless, off-white or light gray crystalline, sparingly water-soluble powder with a molecular weight of 296.54. DSF is a strong chelator of iron, copper, and zinc. It is a stable compound in alkaline media (pH 7.0–9.0), but unstable in acidic media up to pH < 7.0 [10,11].

Only after about 20 years, at the beginning of the 20th century, DSF found application in the rubber industry as an accelerator in the vulcanization process [12].

In 1937, it was noticed that workers exposed to DSF became intolerant to alcohol consumption [13]. In 1951, DSF was approved by the US Food and Drug Administration (FDA) as a drug to treat alcoholism, and for several decades, it was widely used with well-established pharmacokinetic properties, safety, and tolerance. And this is how DSF, under its trade name Antabuse, became one of the most recognizable medicines in the world. DSF is an irreversible inhibitor of aldehyde dehydrogenase (ALDH), an enzyme that converts the first metabolite of ethanol, acetaldehyde, to acetic acid. This leads to the accumulation of acetaldehyde and an unpleasant DSF–ethanol reaction (DER), characterized by, among others, flushing, nausea, vomiting, headache, vertigo, chest pain, cardiac arrhythmia, hypotension, tachycardia, and even death. Typically, after an alcohol intolerance reaction, the patient becomes drowsy (somnolence) and may sleep for several hours. Thus, the DER is the basis for the use of DSF in the treatment of alcoholism. This type of therapy is called aversion therapy: the fear of feeling bad, caused by alcohol consumption during DSF treatment, stops the patient from drinking alcohol. In recent years, many opponents of using DSF in the treatment of patients with alcoholism have appeared in medical circles. They believe that the therapy is unethical because we knowingly expose the patient to the risk of poisoning or even loss of life. There are also arguments that this is inhumane intimidation. Currently, in many countries (but not all), DSF has been withdrawn from treatment. It is worth adding that DSF was the first medication approved to treat people addicted to alcohol. For readers who are more interested in the path that DSF took before becoming a widely known anti-alcohol drug throughout the world, we recommend Kragh’s excellent review paper [10].

Thus, at present, DSF’s career as an anti-alcohol drug is practically coming to an end. However, there have been reports indicating new areas of pharmacological activity of DSF and its metabolites, from which the anticancer activity of this drug is increasingly confirmed, arousing great hopes among doctors and patients.

## 3. Anticancer Activity of DSF

A 38-year-old woman was diagnosed with breast cancer with bone metastases. Bone metastases are defined by oncologists as an unfavorable prognostic factor. The awareness of the irreversibility of the disease process and approaching death caused the patient to become addicted to alcohol. Doctors therefore stopped oncological treatment and began pharmacological treatment of alcoholism, giving the patient a drug that was supposed to discourage her from drinking. The woman died 10 years later (sic!) after a drunken fall from a window. The results of the autopsy were surprising: the bone tumors had disappeared, leaving only a few cancer cells in the marrow. This is a case report from 1971, published by Lewison in 1976 [14]. The drug used to treat women’s alcoholism was, of course, DSF. It is known—based on generally available clinical data—that the average survival period of patients with bone metastases who are not subjected to causal therapy is about 7 months. The described patient lived for another 10 years, and the cause of death was alcoholism, not cancer. The latter—as the description suggests—was defeated by DSF.

Although there had been earlier publications in the 1970s pointing to the anticancer potential of DSF in experimental animals [15,16,17,18], the real breakthrough in DSF’s ‘career’ came only after the aforementioned publication by Lewison [14]. As time passed, interest in the topic increased.

### 3.1. The Basic Mechanisms of Anticancer Activity of Disulfiram

Almost a quarter of a century ago, Hanahan and Weinberg identified six features that characterize virtually all cancers: sustained angiogenesis, evasion of apoptosis, tissue invasion and metastasis, unlimited replicative potential, self-sufficiency in growth signals, and insensitivity to anti-growth signals [19]. It is currently believed that DSF has been shown to directly impact at least three of these traits—inducing apoptosis, acting as an antiangiogenesis agent, and preventing tissue invasiveness and metastasis [20]. DSF has also been shown to exhibit synergy with anticancer drugs [21,22].

As already mentioned, DSF is a symmetrical disulfide (Figure 2). It can be reduced to two molecules of diethyldithiocarbamate (DDC), which are thiols. The metabolic fate of DSF was largely recognized in the 1960s and 1970s [23,24]. Currently, DDC is considered the main biological metabolite of DSF. The destruction of cancer cells observed under the influence of DSF is attributed to the chemical properties of DSF and DDC, which result from their structure. These are mainly two chemical properties, namely the formation of copper complexes and the formation of mixed disulfides.

#### 3.1.1. Mechanism of Anticancer Activity of DSF in the Presence of Copper Ion Cu^2+^

**Figure 3:**
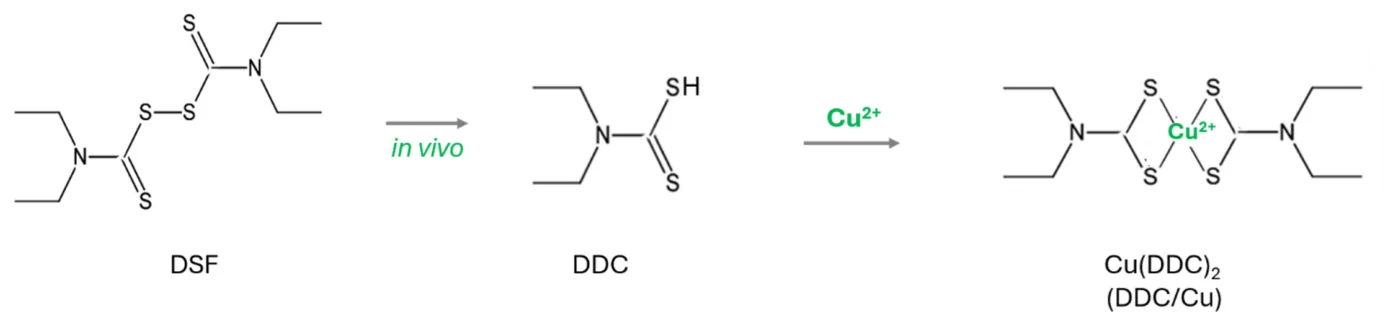
Disulfiram (DSF) reduction to two molecules of diethyldithiocarbamate (DDC) and formation of bis(diethyldithiocarbamate)copper(II) complex, Cu(DDC)_2_ (DDC/Cu).

DSF is a copper ion (Cu^2+^) ionophore. Metal ionophores, similarly to chelators, bind metal ions to form complexes, but unlike chelators, these compounds facilitate the introduction of the metal ion into the cell, thereby increasing its level in the cytoplasmic pool of the cell. Thus, DSF forms a complex with Cu^2+^, increasing intracellular copper ion concentration both in vitro and in vivo, bypassing the need for membrane transporters [25,26]. It has been shown that DSF is rapidly reduced in the body to two molecules of DDC, which, in the presence of Cu^2+^, form bis(diethyldithiocarbamate)copper(II): Cu(DDC)_2_, (DDC/Cu) (Figure 3).

The data suggest that, in cancer cells, the concentration of copper ions is much higher than in normal cells [27]. Many authors claim that copper plays a major role in angiogenesis, a process critical for tumor growth, invasion, and metastasis. Hence, the idea of using metal chelators to bind copper, which reduces its bioavailability in the cell and thus leads to the inhibition of copper-dependent dynamic proliferation and angiogenesis processes in cancer cells [28].

**Figure 4:**
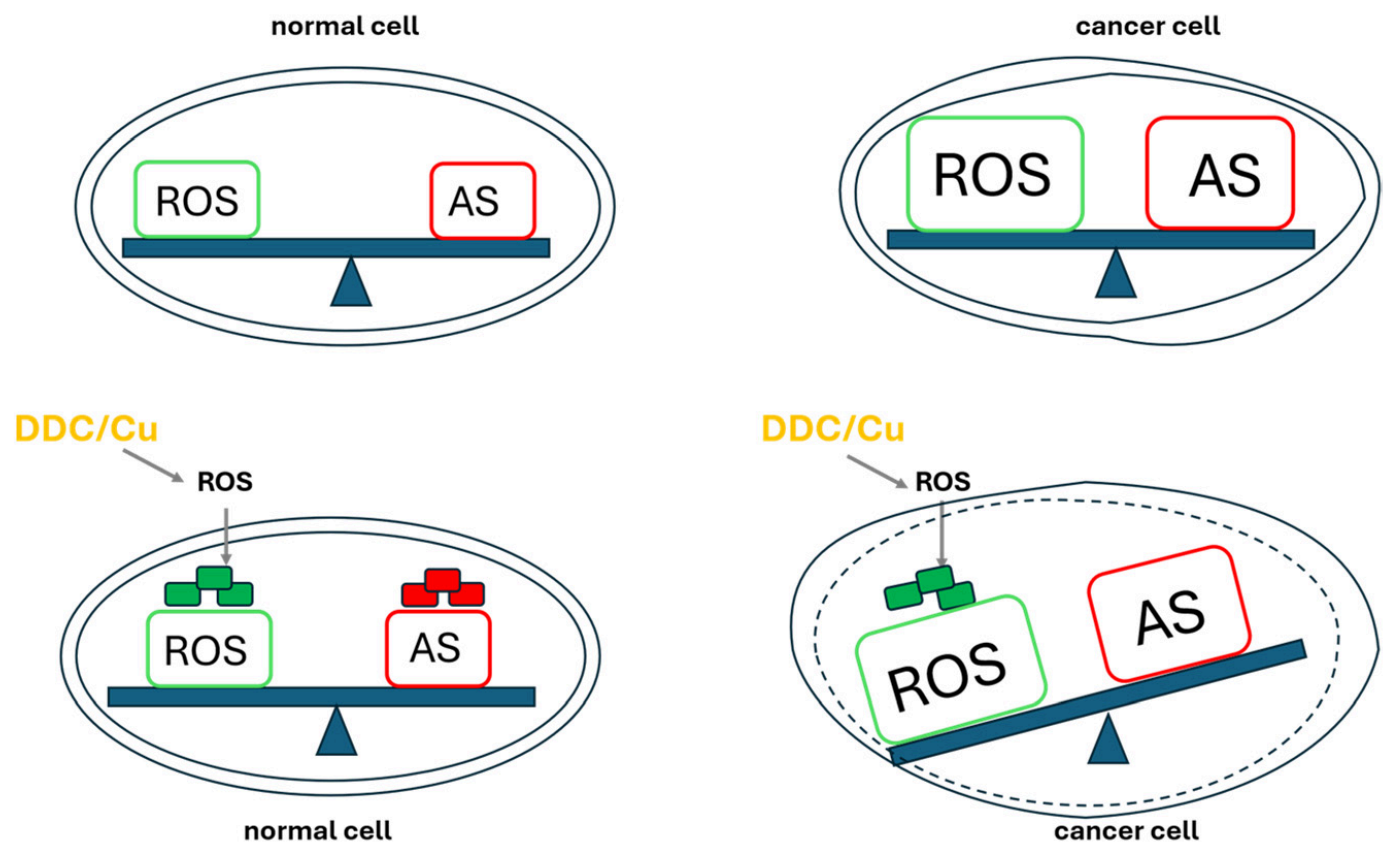
The proposed differential action of DDC/Cu [Cu(DDC)_2_] in normal and cancer cells. Compared to normal cells, cancer cells have higher concentrations of reactive oxygen species (ROS), which contribute to cancer progression. Moreover, the redox balance in cancer cells is regulated by an increased antioxidant system (AS). DDC/Cu [Cu(DDC)_2_], as an oxidative stress inducer, generates additional ROS that exceed the redox capacity in cancer cells, leading to redox homeostasis disruption. Modified from Kim [31].

It is also known that Cu(DDC)_2_ acts as a prooxidant, which means that it is a strong reactive oxygen species (ROS) inducer. It should be noted that the role of ROS in cancer cells seems to be ambiguous. On the one hand, it is known that ROS concentrations in cancer cells are at very high, almost sublethal levels and contribute to cancer progression through a series of interconnected signals. Furthermore, ROS-mediated proliferation signals are correlated with reduced susceptibility of cancer cells to the pro-apoptotic effects of specific anticancer therapies [29,30]. Thus, ROS are oncogenic. Paradoxically, generating ROS in cancer cells, as many drugs do, including Cu(DDC)_2_, is a widely recognized, non-surgical method of treating cancer, due to ROS’s role in triggering cell death. It is also worth noting that this type of therapy is selective and does not harm normal cells [31]. The biochemical basis underlying this phenomenon can be explained by the fact that normal cells have a lower basal level of ROS than cancer cells. ROS in normal cells are essential for cell viability and proliferation. Cancer cells, on the other hand, are in a state of constant oxidative stress. The redox balance in cancer cells is regulated by the increased activity of antioxidant systems, adapted to their metabolic needs [32]. This redox balance in cancer cells can therefore be easily disturbed by exogenous compounds capable of generating ROS and/or weakening the efficiency of the antioxidant system, which consequently leads to exceeding the threshold level of ROS, above which the expression of many genes change, the cell cycle is arrested, transcription factors are activated, and ultimately, cell death is triggered (Figure 4) [31,33,34].

In the opinion of many authors, the key mechanism of the anticancer activity of Cu(DDC)_2_ resulting from the increased level of ROS is the inhibition of the ubiquitin–proteasome system (UPS) [35,36]. This system plays an extremely important role in maintaining cellular homeostasis.

Proteasomes are responsible for the controlled degradation of short-lived signaling proteins and misfolded proteins. The proteins degraded by the UPS include pro-apoptotic proteins and negative regulators of the cell cycle (p53, p21Wf1/Cip1, p27Kip1). Inhibition of the UPS therefore leads to an increase in the level of many important pro-apoptotic factors in the cell. Proteasome inhibition causes IκB protein accumulation, which prevents cytokine-induced activation of the transcription factor inhibitor NF-κB. It should be noted that NF-κB is one of the key factors that promotes cancer survival. Another proteasome substrate is c-myc factor, which leads to increased expression of Fas ligand (FasL). FasL binding to the Fas receptor activates caspase 8, seen as the main caspase of apoptosis, leading inevitably to cell death. The consequence of inhibition of proteasome activity is also an increase in the level of p53 protein, which, in turn, causes an increase in the expression of many pro-apoptotic factors (e.g., Bax) and a decrease in the expression of anti-apoptotic factors. The p53 protein also affects the course of the cell cycle by transactivating p21 protein [37,38,39,40,41].

It is also indicated that the consequence of inhibition of proteasome activity is the activation of a signaling pathway called the unfolded protein response (UPR). It is activated in response to an accumulation of unfolded or misfolded proteins in the lumen of the endoplasmic reticulum (ER). The UPR mechanism aims to be an adaptive response that is designed to reduce stress and restore normal cell function. If this goal is not achieved, the UPR will result in apoptosis. Thus, the course of the UPR determines cell fate, leading to survival or apoptotic death. Zhang et al. indicated that Cu(DDC)_2_ is able to induce apoptosis of breast and pancreatic carcinoma cells via its impact on the ER stress and UPR pathways [42].

Another less frequently discussed aspect of the anticancer activity of DSF involves its role in hydrogen sulfide (H_2_S) metabolism. H_2_S is an endogenous gas that is regarded as an important signaling molecule in the body. The basic role in endogenous H_2_S synthesis is played by three enzymes: cystathionine-γ-lyase (EC 4.4.1.1; CSE), cystathionine-β-synthase (EC 4.2.1.22; CBS), and 3-mercaptopyruvate sulfurtransferase (EC 2.8.1.2; 3-MST). The direct substrates for H_2_S biosynthesis are L-cysteine for CSE, homocysteine for CBS, and 3-mercaptopyruvate for 3-MST. H_2_S performs its signaling role through protein persulfidation (S-sulfhydration). Persulfidation is seen as a reversible post-translational modification. Namely, H_2_S attaches an additional sulfur to the free thiol groups of cysteine residues in its target protein (protein-SH), yielding a hydropersulfide protein (protein–SSH). The protein modified in this way changes its biological activity [43,44,45]. Sen et al. showed that persulfidation of the highly conserved cysteine-38 residue of the p65 subunit of the NF-kappa B increases its ability to bind to ribosomal protein S3 (RPS3), which, in turn, leads to induced anti-apoptotic gene expression [46]. Mustafa et al. indicated that glyceraldehyde-3-phosphate dehydrogenase (GAPDH), a key glycolytic enzyme, is persulfidated at the cysteine-150 residue—the cysteine that is critical for its catalytic activity. It has also been shown that this modification increases GAPDH catalytic activity [47]. It should be recalled that GAPDH plays an important role in the energy metabolism of cancer cells. This means that high activity of GAPDH supports glycolysis of cancer cells and inhibits their death mechanisms [48]. Thus, it is not surprising that endogenously produced H_2_S is viewed as an oncogenic gas, involved in the development of tumorigenesis [49]. It has been proven that the Cu(DDC)_2_ complex is a potent inhibitor of CBS and CSE, enzymes responsible for the synthesis of H_2_S [50]. In this way, the Cu(DDC)_2_ complex, a metabolite of DSF, reduces the production of H_2_S, which protects cancer cells from death.

Thus, the Cu(DDC)_2_ complex exerts anticancer activity through several overlapping mechanisms, among which the key ones are induction of oxidative stress, inhibition of the proteasome, and inhibition of H_2_S synthesis.

Although Cu(DDC)_2_ is frequently described as the principal active anticancer species, current evidence indicates that this conclusion is based mainly on biochemical and animal studies. In humans, the dominance of Cu(DDC)_2_ has not been definitively established, and DSF may exert anticancer effects through several copper-dependent and copper-independent pathways. An example of a copper-independent mechanism of DSF’s anticancer action is the covalent modification of both proteins and low-molecular-weight thiols. This topic is discussed in more detail in the next section.

#### 3.1.2. DSF Acts as a Modifier of Cysteine Residues in Protein and Non-Protein Compounds

**Figure 5:**
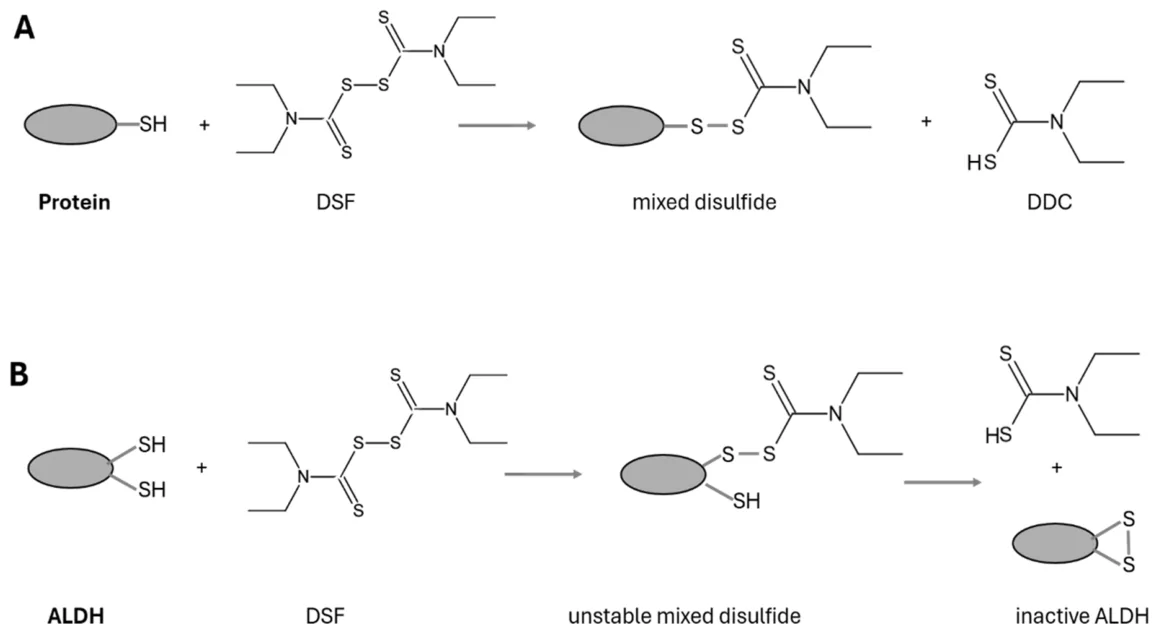
Mechanism of DSF reaction with protein cysteine residues. (**A**) DSF reacts covalently with protein cysteine groups in a thiol–disulfide exchange reaction, forming mixed disulfides. (**B**) In some proteins, like ALDH, inhibition by DSF can lead to the formation of an intramolecular disulfide bond between the active site thiol and the thiol of another cysteine residue via an unstable mixed disulfide adduct. Modified from Shen et al. [52].

DSF reacts covalently with both protein and low-molecular-mass thiols (R-SH), forming dithiodiethylcarbamoyl adducts. In 1981, it was pointed out that this type of reaction was responsible for the fact that “DSF inhibits more or less enzymes and cofactors with −SH groups” [11]. It is now known that DSF reacts covalently with both protein and low-molecular-mass thiols (R-SH), forming mixed disulfides. The mechanism of the reaction between disulfides and thiols is called a thiol–disulfide exchange reaction. It is a fundamental chemical reaction involving the reaction of a thiol group of one chemical compound with a disulfide bond of another chemical compound, resulting in the transfer of disulfide bridges between the molecules [51]. A schematic representation of the reaction of DSF with protein-thiol is shown in Figure 5A.

The reaction product is DDC and a modified protein that could be described as *N*,*N*-diethyldithiocarbamoyl-protein (protein-SS-DDC).

The mechanism of formation of a stable intermolecular mixed disulfide between DSF and the thiol group in the enzyme active site described above is one of the mechanisms of ALDH inhibition by DSF. The second mechanism of ALDH inhibition by DSF is the formation of an intramolecular disulfide bond between the thiol group in the enzyme active site and the thiol group of another cysteine residue via an unstable mixed disulfide adduct [53]. A schematic representation of this reaction of DSF with ALDH is shown in Figure 5B.

Analysis of the ALDH gene superfamily in the databases has revealed that the human genome contains 19 putatively functional genes and three pseudogenes [54]. Thus, aldehyde dehydrogenases (ALDHs) constitute a group of enzymes that catalyze the oxidation of a wide spectrum of aliphatic and aromatic aldehyde substrates to the corresponding carboxylic acids, with NAD(P) serving as an electron-accepting cofactor. Until recently, this pharmacological ALDH and DSF pair, i.e., the enzyme and its inhibitor, was associated mainly with aversion therapy for alcohol-dependent patients. In recent years, ALDH, particularly the ALDH1 isoform, has been identified as one of the most characteristic markers of cancer stem cells (CSCs) [55]. Studies of two murine osteosarcoma cell lines, K7M2 and K12, demonstrated that K7M2 has high metastatic potential, whereas K12 has significantly lower metastatic potential. ALDH activity was also found to be higher in K7M2 cells than in K12 cells [56]. Greco et al. observed a significant reduction in the in vitro growth rate of cells from patients with metastatic osteosarcoma who were treated with DSF [57]. It has also been reported that treatment with this compound decreases spontaneous lung metastatic burden in a syngeneic preclinical model of metastatic murine breast cancer [58]. Thus, the thesis that high ALDH expression in cancer stem cells contributes to tumor initiation, chemotherapy resistance, and metastasis seems undisputed today. However, the mechanism by which ALDH promotes cancer development is not yet fully understood [59]. Recent studies indicate that ALDH participates in the regulation of tumor initiation and progression through the production of retinoic acid (RA). The problem is complex, as it is known that RA can exert a dual effect on cancer, inhibiting or promoting its progression. Colucci et al. draw attention to the aging process of cancer cells. Senescent tumor cells secrete a variety of cytokines and inflammatory factors in the tumor microenvironment (TME), known as the senescence-associated-secretory phenotype (SASP). The authors identified a retinoic acid receptor (RAR) agonist in prostate cancer cells and observed that its reactivation triggers a strong senescent response and tumor-inhibiting SASP [60].

On the other hand, recent research by Fang et al. demonstrated that RA increases immune tolerance, which means weaker recognition of cancer cells [61]. It is worth recalling here that dendritic cells (DCs) are professional promoters of the immune response against tumor antigens. Studies indicate that patients with cancer experience a reduction in DC numbers and impaired function. Therefore, DC vaccination (DCV) was expected to prove an effective therapeutic strategy in cancer treatment. This has been confirmed, and it is now known that DCV is safe, and the induced antitumor responses are well documented in solid tumors [62]. Fang et al. demonstrated that GM-CSF–IL-4-induced differentiating DCs express ALDH1A2 (one of the isoforms of ALDH) and produce RA, which inhibits the maturation of DCs. Genetic knockout of *Aldh1a2* releases and enhances DC function. The authors obtained a similar effect after using a strong inhibitor of ALDH1A2 activity [61]. Thus, RA plays a complex role in cancer progression, both inhibiting and potentially promoting disease progression depending on the biological context.

ALDH is not the only enzyme inhibited by DSF via the thiol–disulfide exchange reaction. In the context of the anticancer activity of DSF, it is worth mentioning glyceraldehyde-3-phosphate dehydrogenase (GAPDH), an important enzyme of the glycolytic pathway [63]. The results of the study by Liberti et al. directly pointed to GAPDH as the “bottleneck” of the Warburg effect. They demonstrated that selective inhibition of GAPDH can cut off the energy supply to aggressive tumors that are highly dependent on glycolysis [64]. Another enzyme inhibited by DSF is 3-phosphoglycerate dehydrogenase (PHGDH). PHGDH catalyzes the first, rate-limiting step in the serine synthesis pathway in humans, which is abnormally expressed in a variety of cancers and contributes to drug resistance [65]. DSF can also impede other key enzymes involved in critical metabolic pathways, including glycolysis, the tricarboxylic acid cycle, and the pentose phosphate shunt [11]. The effect on the activity of metabolic enzymes is also considered when examining the anticancer activity of DSF. However, this topic is beyond the scope of this review.

**Figure 6:**
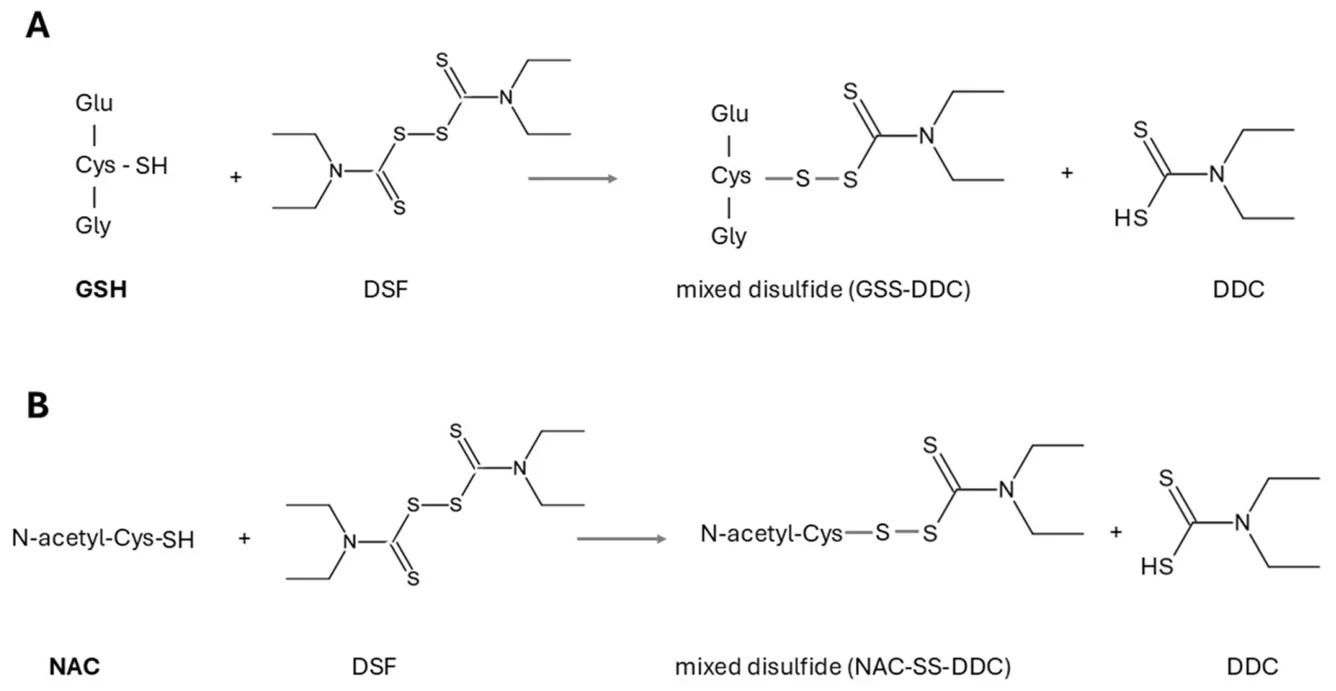
Reaction of DSF with low-molecular-weight thiols: GSH (**A**) and NAC (**B**).

As mentioned earlier, DSF can react not only with protein thiols but also with non-protein thiol compounds. The most common low-molecular-weight thiol compound in nature is glutathione (GSH). Composed of glutamic acid (Glu), cysteine (Cys), and glycine (Gly), it occurs in animal cells in high millimolar concentrations. GSH’s well-known biological functions include antioxidant action and detoxification. This is beneficial for healthy, normal cells but significantly complicates cancer drug therapy. Therefore, much contemporary research focuses on finding ways to reduce the levels of GSH and other antioxidants in cancer tissues. The first and therefore most important paper showing that DSF reacts with GSH was published in 1975 [66]. These observations have been confirmed by other authors [67,68]. A schematic representation of the reaction of DSF with reduced glutathione (GSH) is shown in Figure 6A. DSF is reduced to DDC and the mixed disulfide S-(*N*,*N*-diethyldithiocarbamoyl)-L-glutathione (GSS-DDC) is formed. This process is crucial for cancer cell survival because the reaction between GSH and DSF leads to a decrease in GSH levels in the cell, which reduces the cell’s antioxidant potential and increases the risk of damage, consequently reducing cancer cell survival. The same mechanism accounts for DSF’s ability to react with Cys, an amino acid whose concentration in the cell is a rate-limiting factor for GSH synthesis. Therefore, DSF also contributes to GSH depletion by reducing the concentration of Cys, which is essential for its synthesis.

There is also evidence that DSF’s action extends beyond reducing GSH levels. Mixed disulfides obtained by reacting DSF with the Cys derivative N-acetylcysteine (NAC) (Figure 6B) have also been shown to possess anticancer properties. Lee et al. showed that the occurrence of DNA single-strand breaks in rat liver cells induced by N-nitrosodiethylamine was completely neutralized by pretreatment with NAC–SS–DDC [69]. Studies using Salmonella strains TA98 and TA100 showed that NAC–SS–DDC and GSS–DDC significantly reduce the mutagenic effect of benzo[a]pyrene on these bacterial cells [68].

DSF, as a disulfide, reacts with thiol groups of protein and non-protein compounds, forming dithiodiethylcarbamoyl adducts. Thiol group modification is perceived as a molecular “switch” decisive for the intracellular redox status of thiols and controlling cellular metabolic pathways. The consequence of blocking thiol groups by DSF is cancer cell death.

Thus, DSF exerts anticancer activity by inhibiting not only various ALDH isoforms but also GAPDH and PHGDH. The anticancer activity of DSF is also related to its ability to react with small-molecular-weight compounds such as GSH, Cys, and its derivatives.

### 3.2. Strength of Evidence Across Experimental Systems

The strength of mechanistic evidence supporting DSF’s anticancer activity differs substantially across experimental systems. Several proposed mechanisms are supported primarily by biochemical assays or cell-culture studies, including ALDH inhibition, proteasome disruption, ROS generation, H_2_S modulation, and copper-dependent formation of Cu(DDC)_2_. These findings provide important molecular insights but rely on simplified conditions that may not fully reflect physiological copper availability or tumor microenvironment complexity. In contrast, only a subset of mechanisms has been validated in animal models, such as suppression of cancer stem cell phenotypes, inhibition of tumor growth in xenografts, and enhancement of temozolomide efficacy in glioblastoma. Evidence from human samples remains limited and largely indirect, with clinical studies demonstrating pharmacodynamic effects (e.g., ALDH modulation, copper redistribution) rather than direct mechanistic confirmation. This problem requires further research.

## 4. From Experimental Models to Patients: Sources of Inconsistency in the Activity of DSF

DSF has been found to be ineffective in several clinical cancer trials. Why have DSF’s promising preclinical results not been translated into clinical efficacy? Cell line studies demonstrate its ability to inhibit cancer cell proliferation, migration, and invasion. These studies have also largely elucidated the molecular mechanisms of DSF’s anticancer activity. Yet, clinical trial results remain disappointing [70,71]. The reasons for this translational gap are multifactorial. One of the factors that may contribute to the poor clinical efficacy of DSF is the problem of tumor heterogeneity. This thesis has very strong support in the oncology literature. This phenomenon is described as intratumor heterogeneity (ITH), which leads to the selection of treatment-resistant clones [72,73].

Medical literature also emphasizes that standard “alcoholic” dosing of DSF rarely translates into stable and effective anticancer concentrations in the tumor. This is due to the drug’s very short half-life in the blood and the rapid degradation of its active metabolites in the body [74]. Furthermore, the discrepancy between DSF concentrations achieved in vitro and those that can be safely achieved in the patient’s body is also worth emphasizing. In laboratories, cancer cells are often “flooded” with micromolar concentrations of DSF (where the drug is not subject to rapid metabolism), whereas after oral administration in humans, DSF plasma concentrations are in the nanomolar range. This gigantic, thousand-fold difference means that the mechanisms observed in Petri dishes do not occur in the patient’s body [75].

### 4.1. Disulfiram in the Clinic: A Concise Overview

This section summarizes several studies extracted from published trials evaluating DSF in non-small-cell lung cancer (NSCLC), glioblastoma, and prostate cancer.

Nechushtan et al. conducted a small phase II clinical trial in 15 patients with advanced, metastatic non-small-cell lung cancer (NSCLC) who had previously received standard therapy. Patients received DSF in combination with copper (naturally present in the diet), without additional cytotoxic agents. The treatment was well tolerated, but clinical responses were limited, with disease stabilization noted in some patients and no clear objective responses. The authors suggest that DSF may have biological activity, but its clinical efficacy in NSCLC is insufficient without improved formulation or biomarkers [76].

The phase II clinical trial showed that adding DSF and copper to temozolomide (TMZ) is well tolerated in patients with recurrent, treatment-resistant glioblastoma. However, this therapy demonstrated limited efficacy in the unselected population, with an objective response rate (ORR) of 0% and a 14% clinical benefit rate. The median overall survival was 7.1 months, indicating low activity in re-sensitizing tumors to TMZ [70]. Another clinical trial in patients with recurrent glioblastoma multiforme was conducted by Werlenius et al. This multicenter, open-label, randomized, phase II/III, parallel-group clinical trial was designed to evaluate the efficacy and safety of DSF and copper as adjuvant therapy to alkylating chemotherapy in patients with recurrent glioblastoma multiforme. The obtained results indicated that the therapy used did not improve survival. For example, the median overall survival (OS) was 5.5 months in the group of patients receiving disulfiram and copper vs. 8.2 months in the standard-therapy group. The median progression-free survival (PFS) was 2.3 months vs. 2.6 months, respectively. The authors concluded that the combination of disulfiram and copper with chemotherapy does not provide therapeutic benefit in recurrent glioblastoma multiforme and should not be recommended in this group of patients [77]. The aim of the next clinical trial was to evaluate the safety and efficacy of co-administering DSF with Cu in patients with metastatic, castration-resistant prostate cancer (mCRPC). The authors wanted to determine whether the addition of Cu—necessary for the formation of the active Cu(DDC)_2_ complex—would improve the effect of DSF, as previous clinical trials used DSF as monotherapy. The results showed that oral disulfiram is not effective in the treatment of mCRPC because it is rapidly metabolized to inactive metabolites, and even copper administration does not ensure the formation of the active Cu(DDC)_2_ complex. Due to the lack of treatment efficacy, the study was terminated prematurely. According to the authors, there is a need to develop a stable DSF formulation that will maintain the active drug in circulation [78].

It is worth noting that the cancer stage also influences failure of therapy. Clinical data provide clear evidence: the higher the cancer stage (more metastases, long history of prior treatment, high mutation rate), the less likely it is that traditionally administered DSF will reverse the process. The complexity of advanced tumors outweighs the simple mechanism of action of a single drug.

Thus, the information cited above indicates that the limited clinical efficacy of DSF in cancer patients is primarily attributed to its in vivo pharmacokinetic properties and dependence on copper ions. The lack of predictive biomarkers is also noteworthy. Many authors believe that the full potential of DSF in oncology requires the development of stable, controlled delivery systems that prevent its rapid degradation and enable the formation of the active Cu(DDC)_2_ complex.

A more detailed discussion of this topic is provided in the subsequent sections.

### 4.2. Fast Metabolism and Lack of Active Forms of DSF in the Human Body

The half-life of the currently available oral version of DSF in the blood is approximately 2–4 min [79]. The first metabolite of DSF is DDC, which, in the liver, is rapidly degraded into carbonyl disulphide and dimethylamine or undergoes methylation enzymatically to form S-methyl-DDC [79,80]. The resulting DDC metabolites lack thiol groups and therefore are no longer able to chelate Cu^2+^ ions (Figure 3). As we know, the chemical compound that is mainly responsible for the anticancer effect of DSF is the Cu(DDC)_2_ complex. Let us assume, however, that despite the rapid metabolism of DSF and DDC, some parts of DDC are not degraded into carbonyl disulphide and dimethylamine and are not methylated into the S-methyl-DDC form, and therefore can chelate Cu^2+^. Ions— and this is where the issue of Cu^2+^ ion availability arises. In the human body, the availability of Cu^2+^ ions is tightly regulated, and the concentrations of available Cu^2+^ ions may be insufficient to form the active Cu(DDC)_2_ complex. In preclinical studies, copper supplementation is often used, which significantly enhances the anticancer effect, but in clinical trials, it is not routinely used or is used inconsistently [75].

### 4.3. Lack of Predictive Biomarkers

Without predictive biomarkers, it is difficult to select patients in whom the DSF mechanism of action is likely to work. In other words, we do not know which patients are likely to respond to treatment. The drug’s effectiveness depends on a number of biological variables, such as free copper levels, ALDH1 expression characteristics of CSC, proteasome activity, and individual differences in the metabolism of DSF to its active complexes with Cu^2+^ ions [71,81]. As we know, the active form of the drug is the Cu(DDC)_2_ complex. In some patients, the pool of free copper is low or highly buffered, and there is simply nothing from which to form the active complex. There is no biomarker that can predict who will have sufficient Cu(DDC)_2_ complex concentrations [75]. Another example: ALDH1A1, one of the ALDH1 isoforms, is a marker of CSCs. DSF, as an inhibitor of all ALDH isoforms, should work best in patients with high ALDH1 expression, a large CSC population, and tumors with an ALDH-dependent resistance phenotype. In practice, no clinical trial has selected patients based on ALDH1 expression despite it being a key molecular target of DSF. DSF, as a proteasome inhibitor, should work best in tumors with high proteasome activity or a high burden of misfolded proteins (e.g., myeloma, some breast cancers). However, proteasome activity is not measured in patients, nor are patients selected based on the results of proteotoxic stress measurements.

Thus, because none of these parameters are routinely assessed in patients, clinical trials involve heterogeneous populations in which only a subset of patients have a biological predisposition to respond to therapy. Taken together, DSF is targeted at patients in whom its mechanisms of action are irrelevant [82]. In short, DSF requires a standardized treatment protocol, not “alcoholism-like administration.”

### 4.4. Delivery Systems for DSF

Recent literature highlights the need for advanced formulations such as nanoparticles, liposomes, polymeric microcapsules, or copper-co-delivering systems, which could stabilize DSF, enhance its bioavailability, and ensure controlled formation of the Cu(DDC)_2_ complex within the tumor microenvironment. According to most authors, without such innovations, the therapeutic potential of DSF in oncology is unlikely to be fully realized. Wang et al. developed a poly lactic-co-glycolic acid (PLGA)-encapsulated DSF, protecting DSF from degradation in the bloodstream. The obtained results indicated that PLGA encapsulation extended the half-life of DSF from less than 2 min to 7 h in serum. In in vivo studies using mice, the authors also demonstrated that lung metastasis of tail vein-injected hepatocellular carcinoma (HCC) cells was completely blocked by a combination application of DSF-PLGA and copper gluconate (CuGlu). Cancer nodules and micro-metastases were observed in the control group but not in the treated animals [83]. Liu et al. indicated that liposome-encapsulated DSF (Lipo-DSF) suppressed the growth of MDA-MB-231 human breast cancer (BC) xenografts established by subcutaneous implantation in mice. This effect was dependent on the presence of Cu^2+^. The authors found that, compared to the control group, both Lipo-DSF/CuGlu and Lipo-DSF significantly inhibited tumor growth, but Lipo-DSF/CuGlu demonstrated the highest anticancer efficacy [84].

The comparison of conventional DSF and nano-formulated DSF in anticancer research is summarized in the table below (Table 1).

The comparison between conventional DSF and its nano-formulated counterparts highlights the fundamental reasons why classical DSF has failed to translate into meaningful clinical anticancer activity, despite strong in vitro effects. Conventional DSF is chemically unstable, rapidly degraded to DDC, and highly dependent on the availability of free copper ions, which are tightly regulated in human physiology. As a result, only minimal amounts of the active Cu(DDC)_2_ complex are formed in vivo, leading to weak and inconsistent antitumor effects in animal models and clinical studies. Nanoformulations—such as PLGA nanoparticles, liposomes, and hybrid Cu(DDC)_2_ nanocarriers—address these limitations by stabilizing DSF, protecting it from rapid degradation, and enabling controlled, sustained release. Importantly, several nano-platforms can co-deliver copper, ensuring efficient formation of the active Cu(DDC)_2_ complex directly within the tumor microenvironment. These improvements translate into markedly enhanced antitumor efficacy in animal models, including stronger inhibition of tumor growth, reduced cancer stem cell populations, and improved pharmacokinetic profiles. Although nanoencapsulation is a promising strategy supported by strong preclinical evidence, its necessity for clinical translation remains to be confirmed. Large clinical trials of nano-formulated DSF are still lacking; therefore, conclusions must remain cautious.

## 5. Conclusions and Future Perspectives

Disulfiram (DSF) represents a compelling example of drug repurposing, revealing a broad spectrum of anticancer mechanisms far beyond its traditional use in alcoholism. Preclinical studies consistently demonstrate that DSF and its active metabolite Cu(DDC)_2_ target multiple hallmarks of cancer, including oxidative stress induction, proteasome inhibition, suppression of H_2_S synthesis, and thiol modification of proteins and small molecules. DSF also interferes with key metabolic enzymes such as ALDH, GAPDH, and PHGDH, and shows the ability to eliminate cancer stem cells (CSCs), positioning it as a promising agent for treating resistant and recurrent tumors.

Despite strong mechanistic evidence and robust preclinical efficacy, clinical outcomes remain disappointing. Major translational barriers include the extremely rapid metabolism of DSF, preventing sustained levels of the active compound; limited availability of free Cu^2+^ ions, essential for forming Cu(DDC)_2_; and the absence of predictive biomarkers to identify patients who are most likely to benefit. A critical challenge is the lack of an appropriate drug formulation capable of bypassing DSF’s rapid degradation and ensuring stable delivery of its active complex in vivo. As such, future research should focus on the following:

Developing novel DSF formulations (nanoparticles, liposomes, prodrugs) that stabilize and deliver the active Cu(DDC)_2_ complex;

Standardizing copper supplementation in clinical trials to reproduce preclinical conditions;

Identifying predictive biomarkers, including free copper levels, ALDH1 activity, and proteasome signatures;

Exploring synergistic combinations of DSF with targeted therapies and immunotherapies, particularly in the context of dendritic cell modulation and tumor microenvironment remodeling;

Advancing research on CSC eradication, which may overcome therapeutic resistance.

In conclusion, DSF remains a highly promising anticancer candidate, but its successful clinical translation requires overcoming pharmacokinetic limitations and implementing biomarker-guided patient selection. DSF has come a long way from the rubber industry to oncology. Currently, it remains a highly promising anticancer candidate, but its successful clinical translation requires overcoming pharmacokinetic limitations and implementing biomarker-guided patient selection. It would be overly enthusiastic to claim it will achieve the status of a “wonder drug for cancer,” as such drugs simply do not exist. Nevertheless, further research, primarily concerning the mechanisms of DSF’s biological action and the development of effective delivery technology, could transform this therapy from a promising therapeutic concept into a viable therapeutic option in cancer treatment.

## Figures and Tables

**Table 1:** Comparison of conventional DSF and nano-formulated DSF in anticancer research.

Parameter	Conventional DSF	Nano-Formulated DSF (PLGA Nanoparticles, Liposomes, DSF–Cu Nanocarriers)	References
Stability in plasma	Very low; rapidly degraded to DDC, minimal intact DSF detectable	High; nanoencapsulation protects DSF from rapid degradation and enables controlled release	[74]
Bioavailability	Low and variable; poor tumor penetration	Significantly improved; enhanced tumor accumulation and prolonged circulation time	[85]
Dependence on copper availability	Strong; requires sufficient free Cu to form active DSF–Cu complex	Reduced; nanocarriers can co-deliver Cu, enabling in situ formation of active complex	[75]
Effect on cancer stem cells (CSC)	Limited in vivo activity	Strong inhibition of CSC populations; reduced sphere formation and tumor-initiating capacity	[80]
Antitumor efficacy in animal models	Weak to moderate; requires high doses or Cu supplementation	Strong; marked tumor regression, enhanced apoptosis, improved pharmacokinetics	[80,85]
Targeted delivery to tumor tissue	None	Possible depending on carrier (PLGA, liposomes, hybrid Cu-nanocarriers)	[83,84,86,87,88]
Systemic toxicity	Variable; influenced by rapid metabolism and off-target effects	Lower; controlled release reduces peak toxicity	[85]
Clinical evidence	Several trials, but no consistent anticancer benefit	No large clinical trials yet; promising preclinical data only	[75]

## Data Availability

No new data were created or analyzed in this study. Data sharing is not applicable to this article.

## References

[1] Nosengo N. (2016). Can you teach old drugs new tricks?. Nature.

[2] Kumar R., Harilal S., Gupta S.V., Jose J., Thomas Parambi D.G., Uddin M.S., Shah M.A., Mathew B. (2019). Exploring the new horizons of drug repurposing: A vital tool for turning hard work into smart work. Eur. J. Med. Chem..

[3] Park K. (2019). A review of computational drug repurposing. Transl. Clin. Pharmacol..

[4] Zeng M., Wu B., Wei W., Jiang Z., Li P., Quan Y., Hu X. (2024). Disulfiram: A novel repurposed drug for cancer therapy. Chin. Med. J..

[5] Nakhaei A., Taghavi A., Aliyari M., Afshari A.R., Gheybi E., Jalili-Nik M. (2026). Drug repurposing as a promising therapeutic strategy against renal cell carcinoma. Cancer Chemother. Pharmacol..

[6] Custodio M.M., Sparks J., Long T.E. (2022). Disulfiram: A Repurposed Drug in Preclinical and Clinical Development for the Treatment of Infectious Diseases. Antiinfect. Agents.

[7] Iciek M., Bilska-Wilkosz A., Kozdrowicki M., Górny M. (2023). Reactive Sulfur Species in Human Diseases. Antioxid. Redox Signal..

[8] Biernat-Sudolska M., Rojek-Zakrzewska D., Drożdż K., Bilska-Wilkosz A. (2023). Antimicrobial Activity of *N*,*N*-Diethyldithiocarbamate against *Ureaplasma parvum* and *Ureaplasma urealyticum*. Int. J. Mol. Sci..

[9] Grodzki M. (1881). Über äthylirte Sulfoharnstoffe. Berichte Dtsch. Chem. Ges..

[10] Kragh H. (2008). From Disulfiram to Antabuse: The Invention of a Drug. Bull. Hist. Chem..

[11] Eneanya D.I., Bianchine J.R., Duran D.O., Andresen B.D. (1981). The actions of metabolic fate of disulfiram. Annu. Rev. Pharmacol. Toxicol..

[12] Twiss D., Bazier S.A., Thomas F. (1922). The Dithiocarbamate Accelerators of Vulcanization. J. Soc. Chem. Ind..

[13] Williams E.E. (1937). Effects of alcohol on workers with carbon disulfide. JAMA.

[14] Lewison E.F. (1976). Spontaneous regression of breast cancer. Natl. Cancer Inst. Monogr..

[15] Wattenberg L.W. (1974). Inhibition of carcinogenic and toxic effects of polycyclic hydrocarbons by several sulfur-containing compounds. J. Natl. Cancer Inst..

[16] Wattenberg L.W. (1975). Inhibition of dimethylhydrazine-induced neoplasia of the large intestine by disulfiram. J. Natl. Cancer Inst..

[17] Moriya M., Ohta T., Watanabe K., Watanabe Y., Sugiyama F., Miyazawa T., Shirasu Y. (1979). Inhibitors for the mutagenicities of colon carcinogens, 1,2-dimethylhydrazine and azoxymethane, in the host-mediated assay. Cancer Lett..

[18] Irving C.C., Tice A.J., Murphy W.M. (1979). Inhibition of N-n-butyl-N-(4-hydroxybutyl)nitrosamine-induced urinary bladder cancer in rats by administration of disulfiram in the diet. Cancer Res..

[19] Hanahan D., Weinberg R.A. (2000). The hallmarks of cancer. Cell.

[20] Sauna Z.E., Shukla S., Ambudkar S.V. (2005). Disulfiram, an old drug with new potential therapeutic uses for human cancers and fungal infections. Mol. Biosyst..

[21] Loo T.W., Clarke D.M. (2000). Blockage of drug resistance in vitro by disulfiram, a drug used to treat alcoholism. J. Natl. Cancer Inst..

[22] Wu C.P., Calcagno A.M., Ambudkar S.V. (2008). Reversal of ABC drug transporter-mediated multidrug resistance in cancer cells: Evaluation of current strategies. Curr. Mol. Pharmacol..

[23] Stroemme J.H. (1965). Interactions of disulfiram and diethyldithiocarbamate with serum proteins studied by means of gel-filtration technique. Biochem. Pharmacol..

[24] Gessner T., Jakubowski M. (1972). Diethyldithiocarbamic acid methyl ester. A metabolite of disulfiram. Biochem. Pharmacol..

[25] Allensworth J.L., Evans M.K., Bertucci F., Aldrich A.J., Festa R.A., Finetti P., Ueno N.T., Safi R., McDonnell D.P., Thiele D.J. (2015). Disulfiram (DSF) acts as a copper ionophore to induce copper-dependent oxidative stress and mediate anti-tumor efficacy in inflammatory breast cancer. Mol. Oncol..

[26] Helsel M.E., Franz K.J. (2015). Pharmacological activity of metal binding agents that alter copper bioavailability. Dalton Trans..

[27] Díez M., Arroyo M., Cerdàn F.J., Muñoz M., Martin M.A., Balibrea J.L. (1989). Serum and tissue trace metal levels in lung cancer. Oncology.

[28] Santini C., Pellei M., Gandin V., Porchia M., Tisato F., Marzano C. (2014). Advances in copper complexes as anticancer agents. Chem. Rev..

[29] Nechifor M.T., Neagu T.-M., Manda G. (2009). Reactive oxygen species, cancer and anti-cancer therapies. Curr. Chem. Biol..

[30] Hileman E.O., Liu J., Albitar M., Keating M.J., Huang P. (2004). Intrinsic oxidative stress in cancer cells: A biochemical basis for therapeutic selectivity. Cancer Chemother. Pharmacol..

[31] Kim S.J., Kim H.S., Seo Y.R. (2019). Understanding of ROS-Inducing Strategy in Anticancer Therapy. Oxid. Med. Cell. Longev..

[32] Pelicano H., Carney D., Huang P. (2004). ROS stress in cancer cells and therapeutic implications. Drug Resist. Updat..

[33] Trachootham D., Alexandre J., Huang P. (2009). Targeting cancer cells by ROS-mediated mechanisms: A radical therapeutic approach?. Nat. Rev. Drug Discov..

[34] Wang J., Yi J. (2008). Cancer cell killing via ROS: To increase or decrease, that is the question. Cancer Biol. Ther..

[35] Chen D., Cui Q.C., Yang H., Dou Q.P. (2006). Disulfiram, a clinically used anti-alcoholism drug and copper-binding agent, induces apoptotic cell death in breast cancer cultures and xenografts via inhibition of the proteasome activity. Cancer Res..

[36] Kona F.R., Buac D., Burger A.M. (2011). Disulfiram, and disulfiram derivatives as novel potential anticancer drugs targeting the ubiquitin-proteasome system in both preclinical and clinical studies. Curr. Cancer Drug Targets.

[37] Wu W.K.K., Cho C.H., Lee C.W., Wu K., Fan D., Yu J., Sung J.J.Y. (2010). Proteasome inhibition: A new therapeutic strategy to cancer treatment. Cancer Lett..

[38] Sorokin A.V., Kim E.R., Ovchinnikov L.P. (2009). Proteasome system of protein degradation and processing. Biochemistry.

[39] Maliński M., Cichocki M. (2013). Inhibicja aktywności proteasomu jako nowa strategia w terapii i chemioprewencji nowotworów [Proteasome inhibition as a new strategy in cancer therapy and chemoprevention]. Postepy Hig. Med. Dosw..

[40] Karin M. (2006). Nuclear factor-κB in cancer development and progression. Nature.

[41] Orlowski R.Z. (1999). The role of the ubiquitin-proteasome pathway in apoptosis. Cell Death Differ..

[42] Zhang X., Hu P., Ding S.-Y., Sun T., Liu L., Han S., DeLeo A.B., Sadagopan A., Guo W., Wang X. (2019). Induction of autophagy-dependent apoptosis in cancer cells through activation of ER stress: An uncovered anti-cancer mechanism by anti-alcoholism drug disulfiram. Am. J. Cancer Res..

[43] Andrés C.M.C., Pérez de la Lastra J.M., Andrés Juan C., Plou F.J., Pérez-Lebeña E. (2023). Chemistry of Hydrogen Sulfide-Pathological and Physiological Functions in Mammalian Cells. Cells.

[44] Iciek M., Kowalczyk-Pachel D., Bilska-Wilkosz A., Kwiecień I., Górny M., Włodek L. (2015). S-sulfhydration as a cellular redox regulation. Biosci. Rep..

[45] Paul B.D., Snyder S.H. (2012). H2S signalling through protein sulfhydration and beyond. Nat. Rev. Mol. Cell Biol..

[46] Sen N., Paul B.D., Gadalla M.M., Mustafa A.K., Sen T., Xu R., Kim S., Snyder S.H. (2012). Hydrogen sulfide-linked sulfhydration of NF-κB mediates its antiapoptotic actions. Mol. Cell.

[47] Mustafa A.K., Gadalla M.M., Sen N., Kim S., Mu W., Gazi S.K., Barrow R.K., Yang G., Wang R., Snyder S.H. (2009). H_2_S signals through protein S-sulfhydration. Sci. Signal..

[48] Butera G., Pacchiana R., Mullappilly N., Margiotta M., Bruno S., Conti P., Riganti C., Donadelli M. (2018). Mutant p53 prevents GAPDH nuclear translocation in pancreatic cancer cells favoring glycolysis and 2-deoxyglucose sensitivity. Biochim. Biophys. Acta Mol. Cell Res..

[49] Khattak S., Rauf M.A., Khan N.H., Zhang Q.-Q., Chen H.-J., Muhammad P., Ansari M.A., Alomary M.N., Jahangir M., Zhang C.-Y. (2022). Hydrogen Sulfide Biology and Its Role in Cancer. Molecules.

[50] Zuhra K., Panagaki T., Randi E.B., Augsburger F., Blondel M., Friocourt G., Herault Y., Szabo C. (2020). Mechanism of cystathionine-β-synthase inhibition by disulfiram: The role of bis(*N*,*N*-diethyldithiocarbamate)-copper(II). Biochem. Pharmacol..

[51] Nagy P. (2013). Kinetics and mechanisms of thiol-disulfide exchange covering direct substitution and thiol oxidation-mediated pathways. Antioxid. Redox Signal..

[52] Shen M.L., Lipsky J.J., Naylor S. (2000). Role of disulfiram in the in vitro inhibition of rat liver mitochondrial aldehyde dehydrogenase. Biochem. Pharmacol..

[53] Vallari R.C., Pietruszko R. (1982). Human aldehyde dehydrogenase: Mechanism of inhibition of disulfiram. Science.

[54] Vasiliou V., Nebert D.W. (2005). Analysis and update of the human aldehyde dehydrogenase (ALDH) gene family. Hum. Genom..

[55] Rodriguez-Torres M., Allan A.L. (2016). Aldehyde dehydrogenase as a marker and functional mediator of metastasis in solid tumors. Clin. Exp. Metastasis.

[56] Mu X., Isaac C., Schott T., Huard J., Weiss K. (2013). Rapamycin Inhibits ALDH Activity, Resistance to Oxidative Stress, and Metastatic Potential in Murine Osteosarcoma Cells. Sarcoma.

[57] Greco N., Schott T., Mu X., Rothenberg A., Voigt C., McGough R.L., Goodman M., Huard J., Weiss K.R. (2014). ALDH activity correlates with metastatic potential in primary sarcomas of bone. J. Cancer Ther..

[58] Duan X., Xiao J., Yin Q., Zhang Z., Yu H., Mao S., Li Y. (2014). Multi-targeted inhibition of tumor growth and lung metastasis by redox-sensitive shell crosslinked micelles loading disulfiram. Nanotechnology.

[59] Nguyen A.L., Facey C.O.B., Boman B.M. (2024). The Significance of Aldehyde Dehydrogenase 1 in Cancers. Int. J. Mol. Sci..

[60] Colucci M., Zumerle S., Bressan S., Gianfanti F., Troiani M., Valdata A., D’Ambrosio M., Pasquini E., Varesi A., Cogo F. (2024). Retinoic acid receptor activation reprograms senescence response and enhances anti-tumor activity of natural killer cells. Cancer Cell.

[61] Fang C., Esposito M., Hars U., Byrne R.T., Song B., Huang J., Roichman A., Shue L., Cheng X., Proudfoot J. (2026). Targeting autocrine retinoic acid signaling by ALDH1A2 inhibition enhances antitumor dendritic cell vaccine efficacy. Nat. Immunol..

[62] Hato L., Vizcay A., Eguren I., Pérez-Gracia J.L., Rodríguez J., Gállego Pérez-Larraya J., Sarobe P., Inogés S., Díaz de Cerio A.L., Santisteban M. (2024). Dendritic Cells in Cancer Immunology and Immunotherapy. Cancers.

[63] Taguchi T., Murase S., Miwa I. (2002). Glyceraldehyde metabolism in human erythrocytes in comparison with that of glucose and dihydroxyacetone. Cell Biochem. Funct..

[64] Liberti M.V., Allen A.E., Ramesh V., Dai Z., Singleton K.R., Guo Z., Liu J.O., Wood K.C., Locasale J.W. (2020). Evolved resistance to partial GAPDH inhibition results in loss of the Warburg effect and in a different state of glycolysis. J. Biol. Chem..

[65] Spillier Q., Vertommen D., Ravez S., Marteau R., Thémans Q., Corbet C., Feron O., Wouters J., Frédérick R. (2019). Anti-alcohol abuse drug disulfiram inhibits human PHGDH via disruption of its active tetrameric form through a specific cysteine oxidation. Sci. Rep..

[66] Kitson T.M. (1975). The effect of disulfiram on the aldehyde dehydrogenases of sheep liver. Biochem. J..

[67] Nagendra S.N., Shetty K.T., Subhash M.N., Guru S.C. (1991). Role of glutathione reductase system in disulfiram conversion to diethyldithiocarbamate. Life Sci..

[68] Lee B.H., Bertram B., Rajca A., Frank N., Schmezer P., Wiessler M. (1995). Inhibitory effects of mixed disulfides from disulfiram on the metabolism and genotoxicity of N-nitrosodiethylamine. Arzneimittelforschung.

[69] Lee B.-H., Lee S., Kim Y.-S., Bertram B., Wiessler M. (1996). Mixed disulfides from disulfiram inhibit the benzo[a]pyrene induced mutagenesis. Mutat. Res..

[70] Huang J., Chaudhary R., Cohen A.L., Fink K., Goldlust S., Boockvar J., Chinnaiyan P., Wan L., Marcus S., Campian J.L. (2019). A multicenter phase II study of temozolomide plus disulfiram and copper for recurrent temozolomide-resistant glioblastoma. J. Neurooncol..

[71] Wang L., Yu Y., Zhou C., Wan R., Li Y. (2022). Anticancer effects of disulfiram: A systematic review of in vitro, animal, and human studies. Syst. Rev..

[72] Dagogo-Jack I., Shaw A. (2018). Tumour heterogeneity and resistance to cancer therapies. Nat. Rev. Clin. Oncol..

[73] Pandele A., Whitby A., Lankford S., Macarthur D., Kamaly-Asl I., Barrett D., Grundy R., Kim H., Rahman R. (2024). Multi-Omic Integration Reveal Intra-Tumour Heterogeneity and Identify Disulfiram and Copper as a Synergistic Therapy in Paediatric Ependymoma. Neuro-Oncology.

[74] Skrott Z., Mistrik M., Andersen K.K., Friis S., Majera D., Gursky J., Ozdian T., Bartkova J., Turi Z., Moudry P. (2017). Alcohol-abuse drug disulfiram targets cancer via p97 segregase adaptor NPL4. Nature.

[75] Kannappan V., Ali M., Small B., Rajendran G., Elzhenni S., Taj H., Wang W., Dou Q.P. (2021). Recent Advances in Repurposing Disulfiram and Disulfiram Derivatives as Copper-Dependent Anticancer Agents. Front. Mol. Biosci..

[76] Nechushtan H., Hamamreh Y., Nidal S., Gotfried M., Baron A., Shalev Y.I., Nisman B., Peretz T., Peylan-Ramu N. (2015). A phase IIb trial assessing the addition of disulfiram to chemotherapy for the treatment of metastatic non-small cell lung cancer. Oncologist.

[77] Werlenius K., Kinhult S., Solheim T.S., Magelssen H., Löfgren D., Mudaisi M., Hylin S., Bartek J., Strandéus M., Lindskog M. (2023). Effect of Disulfiram and Copper Plus Chemotherapy vs. Chemotherapy Alone on Survival in Patients with Recurrent Glioblastoma: A Randomized Clinical Trial. JAMA Netw. Open.

[78] Zhang T., Kephart J., Bronson E., Anand M., Daly C., Spasojevic I., Bakthavatsalam S., Franz K., Berg H., Karachaliou G.S. (2022). Prospective clinical trial of disulfiram plus copper in men with metastatic castration-resistant prostate cancer. Prostate.

[79] Johansson B. (1992). A review of the pharmacokinetics and pharmacodynamics of disulfiram and its metabolites. Acta Psychiatr. Scand..

[80] Butcher K., Kannappan V., Kilari R.S., Morris M.R., McConville C., Armesilla A.L., Wang W. (2018). Investigation of the key chemical structures involved in the anticancer activity of disulfiram in A549 non-small cell lung cancer cell line. BMC Cancer.

[81] Zhang S., Zong Y., Chen L., Li Q., Li Z., Meng R. (2023). The immunomodulatory function and antitumor effect of disulfiram: Paving the way for novel cancer therapeutics. Discov. Oncol..

[82] Lu C., Li X., Ren Y., Zhang X. (2021). Disulfiram: A novel repurposed drug for cancer therapy. Cancer Chemother. Pharmacol..

[83] Wang Z., Tan J., McConville C., Kannappan V., Tawari P.E., Brown J., Ding J., Armesilla A.L., Irache J.M., Mei Q.-B. (2017). Poly lactic-co-glycolic acid controlled delivery of disulfiram to target liver cancer stem-like cells. Nanomedicine.

[84] Liu P., Wang Z., Brown S., Kannappan V., Tawari P.E., Jiang W., Irache J.M., Tang J.Z., Armesilla A.L., Darling J.L. (2014). Liposome encapsulated Disulfiram inhibits NFκB pathway and targets breast cancer stem cells in vitro and in vivo. Oncotarget.

[85] Dumbuya I., Pereira A.M., Tolaymat I., Al Dalaty A., Arafat B., Webster M., Pierscionek B., Khoder M., Najlah M. (2024). Exploring Disulfiram’s Anticancer Potential: PLGA Nano-Carriers for Prolonged Drug Delivery and Potential Improved Therapeutic Efficacy. Nanomaterials.

[86] Butcher K., Wang Z., Kurusamy S., Zhang Z., Morris M.R., Najlah M., McConville C., Kannappan V., Wang W. (2024). PLGA-Nano-Encapsulated Disulfiram Inhibits Cancer Stem Cells and Targets Non-Small Cell Lung Cancer In Vitro and In Vivo. Biomolecules.

[87] Xu L., Sun Y., Li Y., Sun J., Guo Y., Shen Q., Wei Q., Shen J.-W. (2022). Disulfiram: A Food and Drug Administration-approved multifunctional role in synergistically drug delivery systems for tumor treatment. Int. J. Pharm..

[88] Shen X., Sheng H., Zhang Y., Dong X., Kou L., Yao Q., Zhao X. (2024). Nanomedicine-based disulfiram and metal ion co-delivery strategies for cancer treatment. Int. J. Pharm. X.

